# Thorakoabdominale Pfählungsverletzung mit Aortenbeteiligung: Fallbericht mit Literaturanalyse

**DOI:** 10.1007/s00113-025-01649-9

**Published:** 2025-10-29

**Authors:** T. Strohmann, P. Schöttes, J. Richter, M. Albert, O. Adamczewski, H. Krahn, J.-P. Stahl

**Affiliations:** 1 Fakultät für Gesundheit, Universität Witten/Herdecke, Witten, Deutschland; 2 Klinik für Unfall‑, Hand- und Wiederherstellungschirurgie, Klinikum Dortmund gGmbH, Münsterstr. 240, 44145 Dortmund, Deutschland; 3 Klinik für Thoraxchirurgie, Klinikum Dortmund, Dortmund, Deutschland; 4 Radiologische Klinik, Klinikum Dortmund, Dortmund, Deutschland; 5 Chirurgische Klinik, Klinikum Dortmund, Dortmund, Deutschland

**Keywords:** Abdominomediastinale Verletzung, Aortendissektion, Endovaskuläre Aortenreparatur, Zwerchfellruptur, Posttraumatische Belastungsstörung, Abdominomediastinal injury, Aortic dissection, Thoracic endovascular aortic repair, Diaphragmatic rupture, Post-traumatic stress disorder

## Abstract

Thorakoabdominelle Pfählungsverletzungen mit Beteiligung der Aorta stellen eine extrem seltene, jedoch hochgradig lebensbedrohliche Traumakonstellation dar. Der vorliegende Fall beschreibt einen 50-jährigen Bauarbeiter, der nach einem Sturz aus etwa 8 m Höhe von einem senkrecht stehenden Holzstiel thorakoabdominell durchbohrt wurde. Dabei kam es zu multiplen Verletzungen von Lunge, Zwerchfell, Milz, Niere sowie zu einer gedeckten Dissektion der thorakalen Aorta. Die Versorgung erfolgte im Rahmen eines interdisziplinären zeitkritischen Managements mit notfallchirurgischen Interventionen und endovaskulärer Aortenreparatur („thoracic endovascular aortic repair“ [TEVAR]). Trotz initialen hämorrhagischen Schocks und beatmungsassoziierten Komplikationen konnte der Patient stabilisiert und nach 6 Wochen in die stationäre Rehabilitation überführt werden. Der Fall unterstreicht die Bedeutung strukturierter Rettungsketten, moderner endovaskulärer Techniken sowie einer frühzeitigen psychotraumatologischen Betreuung zur Verbesserung des Langzeitergebnisses und zur Vermeidung posttraumatischer Folgestörungen.

## Abstract (en)

Thoracoabdominal impalement injuries involving the aorta represent an extremely rare but highly life-threatening trauma scenario. We report the case of a 50-year-old construction worker who sustained an impalement injury after falling approximately 8m onto an upright standing wooden hammer handle, resulting in thoracoabdominal penetration. The injury resulted in multiple trauma, including damage to the lungs, diaphragm, spleen and kidneys as well as a contained dissection of the thoracic aorta. The patient was treated within the framework of an interdisciplinary, time-critical management approach involving emergency surgical interventions and thoracic endovascular aortic repair (TEVAR). Despite initial hemorrhagic shock and respiratory complications, the patient was successfully stabilized and transferred to inpatient rehabilitation after 6 weeks. This case highlights the importance of structured emergency response systems, advanced endovascular techniques and early psychological trauma care to improve long-term outcomes and prevent posttraumatic stress disorders.

## Einleitung

Thorakale und thorakoabdominale Pfählungsverletzungen treten sehr selten auf und sind typischerweise Folge ungewöhnlicher Unfallmechanismen. Im Gegensatz zu stumpfen Thoraxtraumata folgen sie häufig komplexen Verletzungsmustern mit Beteiligung intrathorakaler und intraabdomineller Strukturen wie Lunge, Herz, Aorta und viszeralen Organen [1].

Penetrierende Thoraxverletzungen machen in industrialisierten Ländern lediglich 3–10 % aller Thoraxtraumata aus [1]; nur wenige Betroffene erreichen lebend ein Traumazentrum. Die Kombination vaskulärer thorakaler Verletzungen mit zusätzlichen Organläsionen geht mit einer signifikant erhöhten Mortalität einher [2]. Im Folgenden wird ein Fall mit gedeckter Aortenruptur infolge einer thorakoabdominalen Pfählung vorgestellt, der erfolgreich interdisziplinär versorgt werden konnte.

## Fallbericht

### Unfallmechanismus und Verletzungsmuster

Ein 50-jähriger Bauarbeiter stürzte während Kanalarbeiten aus einer Höhe von ca. 8 m in einen Schacht. Dabei kam es zur Pfählung durch einen senkrecht stehenden Vorschlaghammerstiel, der den Körper von links kaudal im Milzbereich durchdrang und rechts dorsoapikal im Bereich der Skapula austrat.

### Präklinische Versorgung und Transport

**Abb. 1:**
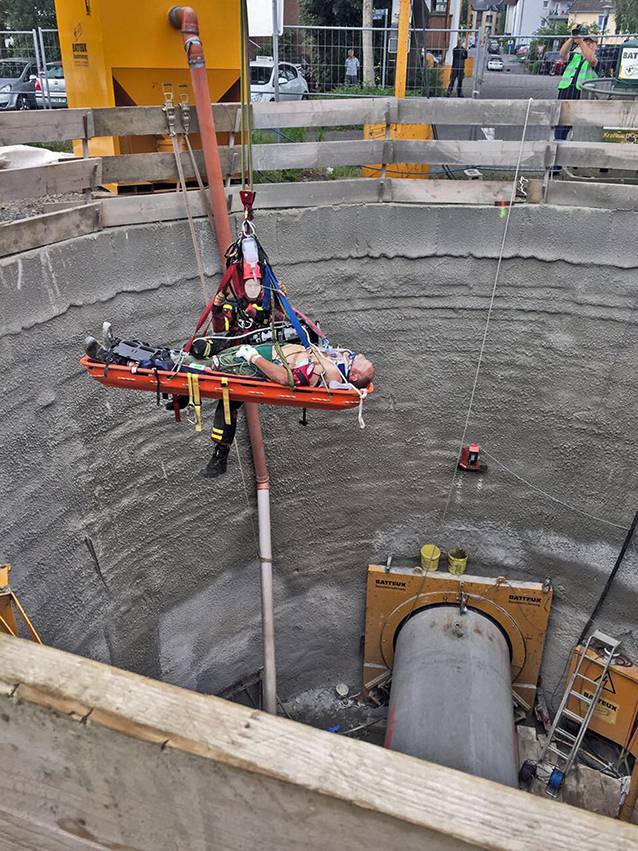
Rettung per Kran-Seilwinde aus dem Bauschacht – intubiert und beatmet

Die Erstversorgung unter erschwerten Bedingungen im engen Schacht beinhaltete eine umgehende Intubation, beidseitige Bülau-Drainagen-Anlage sowie eine hämodynamische Stabilisierung. Für die Bergung wurde das externe Stielende gekürzt. Es erfolgten die technische Bergung durch ein zusätzliches Team der Höhenrettung (Abb. 1) und im Anschluss die Verlegung per Hubschrauber in ein überregionales Traumazentrum. Die Zeitspanne vom Unfallereignis bis zur Schockraumaufnahme betrug 126 min.

### Schockraummanagement und Bildgebung

**Abb. 2:**
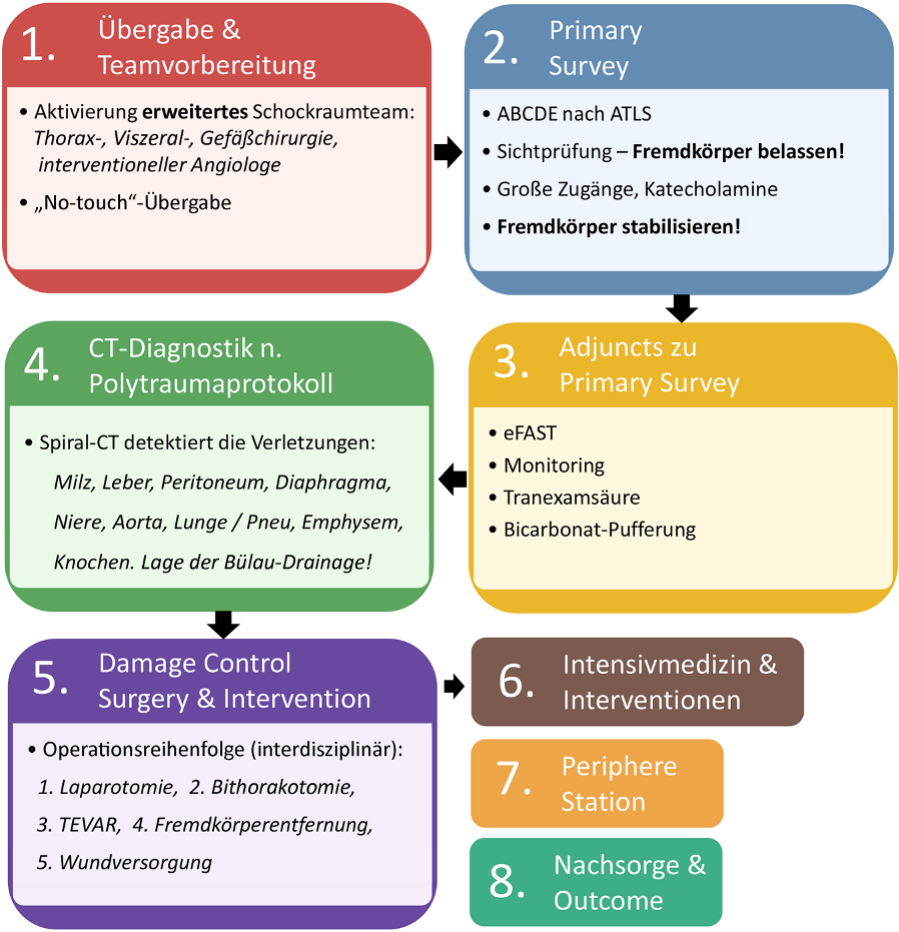
Flowchart der Schockraumstrategie für thorakoabdominelle Pfählungsverletzungen mit Aortenbeteiligung und nachfolgende Behandlungsschritte

**Abb. 3:**
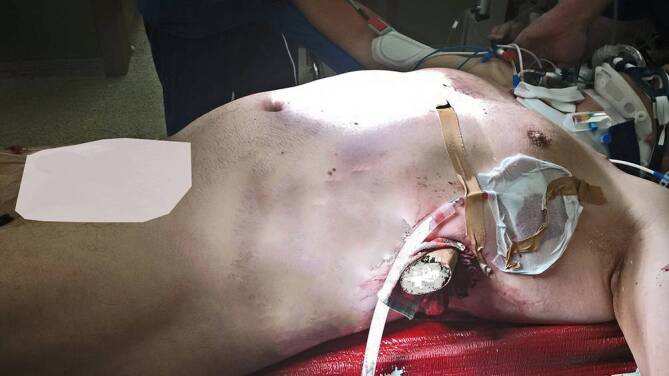
Lagerung im Schockraum. Ansicht von links. Holzpfahl noch in situ

**Abb. 4:**
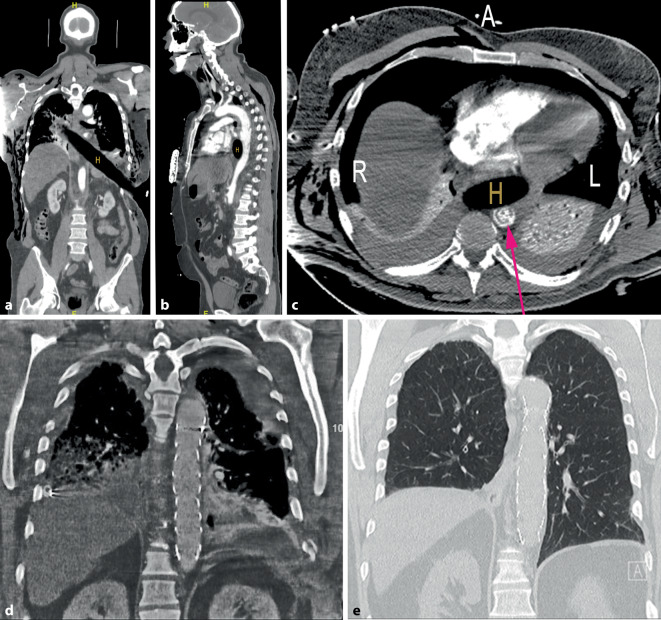
**a–e** Computertomographische Darstellungen. **a–c** zeigen am Unfalltag die Lagebeziehung zwischen dem Holzstiel (*H*) und den Organen. Die Aorta ist durch den anliegenden Fremdkörper verletzt und verlagert. **d** zeigt 10 Tage nach Unfall die Aorta mit Stent. **e** CT-Thorax 7 Jahre später. Das Lungenparenchym hat sich restrukturiert. Zwerchfellhochstand rechts und fehlende Milz links. Keine Diaphragma-Hernie. *R* rechte und *L* linke Thoraxseite, *A* anterior, *H* Holzpfahl. *Pfeil* Aorta descendens mit Dissektionsmembran

Nach der Schockraumankündigung hielt sich ein erweitertes Team (Abb. 2) dort bereit. Bei Aufnahme präsentierte sich der Patient hämodynamisch instabil (Abb. 3). Die Polytrauma-Computertomographie (Abb. 4a–c) offenbarte eine transmediastinale Pfählungsverletzung (H) mit Verlagerung von Herz und Aorta, zwei kurzstreckige Dissektionen (*roter Pfeil*) der Aorta descendens (Typ IIIa nach DeBakey bzw. Typ B nach Stanford), eine rechtsseitige Lungenkontusion mit linksseitigem Pneumothorax (Fehllage der Drainage), Rippenserienfrakturen beidseits, ein ausgedehntes Weichteilemphysem, eine mehrfragmentäre rechtsseitige Skapulafraktur, eine Milzruptur mit aktiver Blutung, eine Zwerchfellruptur sowie kleinere Lazerationen der linken Niere.

### Operatives Vorgehen

Die notfallmäßige interdisziplinäre Operation gliederte sich in mehrere Schritte: Im Rahmen einer Laparotomie erfolgten die Splenektomie sowie die Versorgung abdomineller Peritonealdefekte. Dann diente eine bilaterale Thorakotomie der Resektion verletzter Lungenanteile. Danach wurde die A. femoralis freigelegt und ein thorakaler endovaskulärer Stent-Graft (Valiant 30/30/156) zur Stabilisierung der Aortendissektion implantiert. Erst nach erfolgreicher Stentplatzierung entfernt das Team den Fremdkörper. Anschließend erfolgten die Rekonstruktion des Zwerchfells und die Wundversorgung. Die Operationsdauer betrug etwa 7 h.

### Intensivmedizin und Verlauf

Intra- und unmittelbar postoperativ war neben Katecholamingaben eine Massentransfusion erforderlich: 12 Erythrozytenkonzentrate, 14 Fresh-Frozen-Plasma, ein Thrombozytenkonzentrat, 2 g Tranexamsäure, 2 g Fibrinogen. Die Thorax- und Lungenschädigung behandelten wir neben beidseitigen Bülau-Drainagen durch ein prolongiertes Beatmungsschema. Ab dem zweiten Tag erfolgte die Beatmung via Tracheotomie. In der Kontroll-Computertomographie (CT) zeigte sich ein persistierender linksseitiger Pneumothorax ohne Mediastinalverlagerung, eine stabile Stentposition ohne Endoleak, Infiltrate im linken Unterlappen sowie eine dorsobasale rechtsseitige Pneumonie (Abb. 4d). Die respiratorische Situation verbesserte sich unter korrigierter Drainagenpositionierung, angepasster Antibiotikatherapie und differenzierter Beatmung sowie Lagerungsmaßnahmen. Eine weitere Komplikation stellte eine Wundheilungsstörung dar, die chirurgisch saniert wurde. Nach 3 Wochen konnte der Patient extubiert und auf die periphere Station verlegt werden.

Ab der vierten Woche berichtete er über psychotraumatypische Symptome: Er litt unter starken Ängsten, Einschlafstörungen und „sah“ wiederholt Bilder seines Unfalls mit der erlebten Atemnotsituation. Er verneinte jedoch Flashback-Symptome. Das Psychologenteam der Klinik wurde hinzugezogen: Edukative Gespräche und Bewältigungsstrategien im Sinne der kognitiven Verhaltenstherapie sowie der Traumapsychotherapie (z. B. Erlernen von Entspannungstechniken und Einschlaferleichterung sowie Emotionsregulation u. a.) halfen. Anxiolytische Medikamente ergänzten die Behandlung.

### Outcome und Nachsorge

**Abb. 5:**
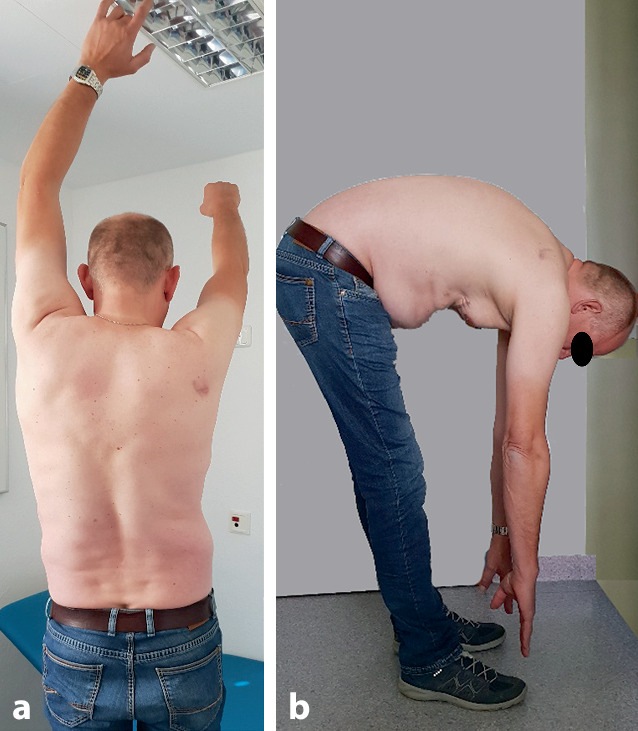
Funktionelle Nachuntersuchung 2 Jahre nach Trauma: **a** Freie Schulterbeweglichkeit links, rechts 120 Grad Elevation. **b** Rumpfbeugung mit Finger-Boden-Abstand 10 cm

Nach sechs Wochen konnte die Verlegung in die stationäre Rehabilitation erfolgen. Arbeitsfähigkeit erreichte der Patient 18 Monate nach dem Traumaeintritt. Zwei Jahre nach der Verletzung waren die Werte für Spirometrie und Bodyplethysmographie noch auffällig. So betrugen die Vitalkapazität (VC) nur 3,7 l (= 63 % der Norm) und die totale Lungenkapazität nur 6,2 l (= 72 % der Norm). Die posttraumatischen Veränderungen im Lungenparenchym entsprachen einer mittelgradigen restriktiven Ventilationsstörung. Der geringe vorbestehende Zwerchfellhochstand rechts hatte sich um ca. 2 Querfinger vergrößert. Im Rahmen des Rentengutachtens wurden als Dauerfolgen eine belastungsabhängige Kurzatmigkeit, eine mittelgradige restriktive Ventilationsstörung sowie eine rechtsseitige Zwerchfellhochstellung mit eingeschränkter Zwerchfellbeweglichkeit beschrieben. Nach der Brustkorb- und Skapulafraktur erreichte er eine aktive Elevation und Abduktion von 140° mit der rechten Schulter (Abb. 5). Die Gesamt-MdE wurde mit 50 % angesetzt. Eine Thorax-CT-Kontrolle 7 Jahre später dokumentierte eine gute Regeneration der betroffenen Lungenflügel und der Aorta (Abb. 4e).

## Diskussion

Thorakoabdominelle Pfählungsverletzungen mit Aortenbeteiligung stellen eine hochkomplexe und seltene Verletzungskonstellation mit potenziell letalem Verlauf dar [3–6]. Der vorliegende Fall demonstriert die Notwendigkeit eines strukturierten interdisziplinären Managements (vgl. Abb. 2), insbesondere bei multiplen Organverletzungen.

### Vergleich mit publizierten Fällen

Viele Patienten mit einer Zweihöhlenverletzung und gleichzeitiger Aortenbeteiligung versterben präklinisch. Die gezielte Literaturrecherche (Tab. 1) ergab nur 7 vergleichbare Fallberichte aus den letzten 37 Jahren. Die Bedeutung einer strukturierten präklinischen sowie operativen Versorgung wird in der Literatur übereinstimmend betont [1–7]. Besonders auffällig im Vergleich ist, dass in unserem Fall keine offene thorakale Aortenrekonstruktion erforderlich wurde. Der Einsatz einer endovaskulären Stent-Prothese (TEVAR) war entscheidend für die Kreislaufstabilisierung und die Kontrolle der potenziellen Blutungsquelle im Rahmen der Fremdkörperentfernung [8–10]. Die moderne endovaskuläre Therapie ermöglicht eine signifikante Reduktion der Mortalität auf etwa 6–10 % [11, 12].

**Tab. 1:** Literatur zu kombinierten thorakalen Pfählungen mit Aortenverletzung

Autoren	Trauma	Beteiligte Strukturen	Diagnostik	Therapie	Outcome	Besondere Aspekte
Fradet et al. (1988) [1]	Armbrustbolzen	Aorta, Leber, Zwerchfell	CT	Thorakotomie, Patch-Plastik der Aorta	Überlebt	Bedeutung von präoperativer CT betont
Noguchi et al. (1991) [2]	Verkehrsunfall	Aorta descendens (Transsektion)	Klinische Diagnose, Röntgen	Thorakotomie, Primärnaht	Überlebt	Schnelle Notfallintervention entscheidend
Kipfer und Carrel (2000) [3]	Messerstich	Aorta descendens, kein Lungenbefall	CT	Thorakotomie mit extrakorporaler Zirkulation	Überlebt	Kardiopulmonaler Bypass für sichere Aortennaht
Chittihavorn et al. (2008) [4]	Stabpfählung	Aorta descendens, kein Lungenschaden	Röntgen, Drainage, CT	Thorakotomie, Aorteninterponat	Überlebt	Fremdkörperfixierung für den Transport verhindert Komplikationen
Lau et al. (2009) [5]	Stachelrochenstich	Aorta ascendens, Herz, V. cava	Klinische Diagnose	Notfallthorakotomie unter fortgeführter Reanimation	Verstirbt	Kombination von mechanischem und toxischem Schaden
Tsuei et al. (2009) [6]	Holzpfahlpfählung	Aortenbogenkontusion ohne Perforation	Arteriogramm, Venogramm, Ösophagramm	Thorakotomie, Lungenkeilresektion, Thoracic duct ligation	Überlebt	Anatomische Variante („bovine arch“) schützte vor Gefäßperforation
Thomson und Knight (2010) [7]	Holzstab Pfählung bilateral	Lunge, Aortakontusion, Zwerchfell, Magen, Leber	CT	Thorakotomie beidseits	Überlebt	Bilaterale perforierende und Kontusionsverletzung

### Psychologische Aspekte

Der frühzeitige Einsatz einer psychotraumatologischen Unterstützung hatte einen wesentlichen Einfluss auf den Rehabilitationsverlauf und die Entwicklung von schwerwiegenden traumatypischen Symptomen verhindern können. Während der Patient später berichtete, unmittelbar nach dem Sturz bei vollem Bewusstsein froh gewesen zu sein, dass nach seinen eigenen Worten „*ja glücklicherweise nichts Schlimmes passiert*“ sei, änderte sich sein Gefühlsempfinden angesichts des lebensbedrohlichen Verletzungsmusters durch die empfundene Bedrohungssituation eklatant. Der Verletzte sagte dazu später selbst: „*Es reicht nicht aus, wenn nur mein Körper geheilt wird …*“. Nach solch dramatischen Erlebnissen ist mit einer posttraumatischen Belastungsstörung (PTBS) zu rechnen. Daher gehört die frühzeitige Einbindung einer psychologischen Unterstützung zum primären Behandlungskonzept. Darüber hinaus sollte in den ersten 6 Monaten ein regelmäßiges Monitoring auf psychische Belastungssymptome (Ängste, Schlafstörungen, Albträume u. a.) erfolgen. In der bisherigen Literatur wird dieser Aspekt selten thematisiert, gewinnt jedoch zunehmend an Bedeutung. Die Psychotraumatherapie, besonders die evidenzbasierte Methode Eye Movement Desensitization and Reprocessing (EMDR), hat in diesem Kontext einen festen Stellenwert [13].

### Limitationen

Als Einzelfalldarstellung ist die Generalisierbarkeit eingeschränkt. Zudem ist die Übertragbarkeit des Managements abhängig von den verfügbaren Ressourcen und den interdisziplinären Strukturen des jeweiligen Versorgungszentrums. Es fehlen Langzeitdaten vergleichbarer Fälle aus der Literatur.

## Kernaussagen für die Praxis

- Thorakale Pfählungsverletzungen resultieren aus ungewöhnlichen Unfallmechanismen.
- Die Kombination aus Aortenverletzung und Mehrorgantrauma erfordert ein interdisziplinäres zeitkritisches Vorgehen und ein erweitertes Schockraumteam.
- Die Stabilisierung und Belassung des Fremdkörpers bis zur operativen Entfernung vermeiden sekundäre Komplikationen.
- Die endovaskuläre Versorgung mittels TEVAR sollte bei gedeckten Aortenrupturen als primäre Therapieoption angestrebt werden.
- Ein psychologisches Monitoring auf Belastungssymptome und frühzeitige psychotraumatologische Interventionen verbessern die Langzeitprognose signifikant.

## References

[1] Fradet G, Nelems B, Müller NL (1988) Penetrating injury of the torso with impalement of the thoracic aorta: preoperative value of the computed tomographic scan. Ann Thorac Surg 45:680–681. 10.1016/S0003-4975(10)64779-0 3377582 10.1016/s0003-4975(10)64779-0

[2] Noguchi K, Sudo K, Kodama J et al (1991) A case report of emergency surgical repair of traumatic transection of thoracic descending aorta. Kyobu Geka 44:965–968 1942696

[3] Kipfer B, Carrel TP (2000) Penetrating stab injury of the thoracic aorta. Circulation 102:1068. 10.1161/01.cir.102.9.1068 10961974 10.1161/01.cir.102.9.1068

[4] Chittithavorn V, Rergkliang C, Chetpaophan A et al (2008) A nonfatal impalement injury of the thoracic descending aorta: a case report. J Trauma 64:E1–3. 10.1097/01.ta.0000233911.05113.25 17514059 10.1097/01.ta.0000233911.05113.25

[5] Lau HK, Chua ISY, Ponampalam R (2020) Penetrating Thoracic Injury and Fatal Aortic Transection From the Barb of a Stingray. Wilderness Environ Med 31:78–81. 10.1016/j.wem.2019.09.004 31983600 10.1016/j.wem.2019.09.004

[6] Tsuei MK, Riley RD, Oaks TE et al (2001) Mediastinal Impalement with Survival: A Case Report. Am Surg 67:594–596. 10.1177/000313480106700618 11409811

[7] Thomson BN, Knight SR (2000) Bilateral thoracoabdominal impalement: avoiding pitfalls in the management of impalement injuries. J Trauma 49:1135–1137. 10.1097/00005373-200012000-00029 11130503 10.1097/00005373-200012000-00029

[8] Brown CVR, de Moya M, Brasel KJ et al (2023) Blunt thoracic aortic injury: A Western Trauma Association critical decisions algorithm. J Trauma Acute Care Surg 94:113–116. 10.1097/TA.0000000000003759 35999667 10.1097/TA.0000000000003759

[9] Mazzaccaro D, Righini P, Fancoli F et al (2023) Blunt Thoracic Aortic Injury. J Clin Med. 10.3390/jcm12082903 37109240 10.3390/jcm12082903 PMC10142366

[10] Shelile L, Ngema S, Kgopane T et al (2025) Hybrid management of a 13-year-old boy with a descending thoracic aortic injury from an impaled knife. J Vasc Surg Cases Innov Tech 11:101740. 10.1016/j.jvscit.2025.101740 40046308 10.1016/j.jvscit.2025.101740 PMC11879667

[11] Rousseau H, Dambrin C, Marcheix B et al (2005) Acute traumatic aortic rupture: a comparison of surgical and stent-graft repair. J Thorac Cardiovasc Surg 129:1050–1055. 10.1016/j.jtcvs.2004.12.023 15867779 10.1016/j.jtcvs.2004.12.023

[12] Deutsche Gesellschaft für Gefäßchirurgie und Gefäßmedizin e. V. (DGG) (2021) Leitlinie S2K Typ B Aortendissektion (004-034). AWMF-Register, Berlin. URL: https://register.awmf.org/de/leitlinien/detail/004-034

[13] Richter J (2022) Schmerz sucht Ursache: Neue Wege in der Schmerztherapie – mit Therapieempfehlungen und begleitenden Übungen. 1. Aufl. Springer Berlin Heidelberg. ISBN 978-3662649039

